# Alcohol Use and Implications for Public Health: Patterns of Use in Four Communities

**DOI:** 10.4103/0970-0218.66875

**Published:** 2010-04

**Authors:** N Girish, R Kavita, G Gururaj, Vivek Benegal

**Affiliations:** National Institute of Mental Health and Neurosciences, Bangalore – 560 029, India

**Keywords:** Alcohol, patterns of use, harmful drinking, transitional areas, public health, policy

## Abstract

**Background:**

Alcohol is one of the leading causes of death and disability globally and in India. Information on quantum and pattern of consumption is crucial to formulate intervention programs.

**Objectives:**

To identify the extent and pattern of alcohol use in urban, rural, town and slum populations using a uniform methodology.

**Materials and Methods:**

Door-to-door survey was undertaken and simple random sampling methodology was adopted; households were the primary sampling unit. One respondent in each alcohol-user household was randomly chosen for detailed interview.

**Results:**

Overall, 13% of males and females consumed alcohol. Proportion of users was greater in town (15.7%) and among 26–45 years (67.4%). Whisky (49%) and arrack (35%) were the preferred types and the preferences differed between rural (arrack) and urban (beer) areas. Nearly half (45%) of rural population were very frequent users (consuming daily or every alternate-days) as against users in town (23%) or slum (20%). Two-thirds were long-term users and the proportions were greater in the rural and town areas. While, overall 17% of the users were heavy-users, frequent-heavy-drinking was more in slum and rural areas. Nearly two-thirds consumed alcohol in liquor-shops, restaurants, bars and pubs. Habituation and peer-pressure were the key reasons for alcohol use.

**Conclusions:**

The study documented alcohol use and patterns of use in four different communities particularly in transitional areas using similar methodology. Many of the patterns identified are detrimental to health both immediate and over the long period of time.

## Introduction

Alcohol is one of the leading causes of death and disability globally. About two billion people worldwide consume alcoholic beverages and one-third (nearly 76.3 million) is likely to have one or more diagnosable alcohol use disorders.(1) Alcohol is attributed to nearly 3.2% of all deaths and results in a loss of 4% of total DALYs (58 million).(2) It is acknowledged that countries which had low alcohol consumption levels are now witnessing an increasing consumption pattern.(1) WHO estimates for the South East Asian countries indicate that one-fourth to one-third of male population drink alcohol(3) with increasing trends among women.(4)

In India, the estimated numbers of alcohol users in 2005 were 62.5 million, with 17.4% of them (10.6 million) being dependant users(5) and 20–30% of hospital admissions are due to alcohol-related problems.(6) Few studies have documented the pattern and profile of alcohol use and its impact in hospital- and population-based settings.(7,8) Variations in consumption patterns in different socioeconomic groups are vital to formulate clearly defined strategies for both demand and supply reduction and to organize required services in general and specific populations. Hence, the present study was undertaken to identify the extent and pattern of alcohol use in four different geographic settings of urban, rural, town and slum populations in Bangalore using a similar methodology.

## Materials and Methods

The present study was part of the larger study: “Burden and socio-economic impact of alcohol use.”(9) It was a cross-sectional study undertaken in four distinct geographic settings (urban, rural, slum and town) of Bangalore district in India. A simple random sampling methodology was adopted. For the rural component, five large villages (having atleast 750–1000 households) in Kanakpura Taluka, Bangalore urban district were identified. Five wards from Kanakpura town were chosen to include town population. One large slum (Sriramapuram slum) was chosen randomly from among the registered slums of Bangalore. To cover the urban population, one ward in the southern part of Bangalore city with a predominantly middle class population was chosen. Commercial establishments were excluded from the survey in all the four areas during survey time.

The primary sampling unit was the individual household. Trained research staff used pre-tested semi-structured questionnaire to collect information. Preliminary information was collected from a responsible adult (being aware of all family details) of the household. Focus of information gathering was on socio-demographic characteristics and alcohol use among individual adult male and female members. Any individual with history of alcohol use in the 12 months prior to the date of survey was considered as an alcohol user for the purpose of this study.(10) Each household was classified as an alcohol user or alcohol non-user household for the purpose of this study.

One respondent in each alcohol-user household was randomly chosen (by lottery method) for detailed interview. Female alcohol users, whenever available, were preferentially interviewed. Information pertaining to type, frequency, quantity, duration and context of alcohol use was gathered. Drinking patterns were classified as harmful drinking, hazardous drinking and pathological drinking based on data collected by direct interviews with the selected respondent. The users were also classified as light (less than five drinks in one sitting) or heavy users (more than five drinks in one sitting) and frequent (consuming at least once a week to daily or nearly daily basis) or infrequent drinkers (consuming at least once a month to once or twice in a year). The current paper focuses and characterizes the pattern of alcohol use in four geographical settings of Bangalore district.

## Results

**Figure 1:**
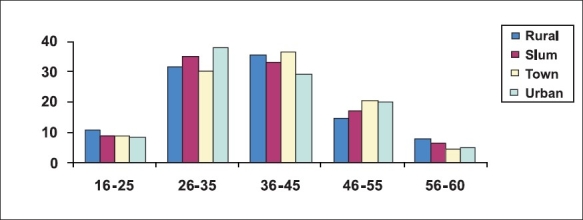
Age distribution of the users

A total of 28,507 individuals were enumerated from among 6,997 households. The overall response rate was 88.5%. In the total population enumerated, during the one year reference period, 13% had consumed alcohol (23.7% males and 1.5% females) and 3250 households (46%) were alcohol user households. Proportions consuming alcohol were greater in the transitional town population (males 28.1% and females: 2.3%). More than two-thirds of the users were in the 26–45 years age–group and 10% were adolescents and youth (16–25 years of age) [Figure 1].

Nearly half (43%) of the users in rural areas were illiterate. Nearly three-fourths (71%) of urban users had completed at least high school and these proportions was less among slum and town populations. Majority of the rural alcohol users were either unskilled laborers or farmers and large number of urban users were either skilled workers or professionals. Nearly 40% of the study population reported an income below the poverty line; 65% of rural alcohol users reported an income twice the poverty level incomes.

## Alcohol use

Type of alcohol [Table 1]: Among the total users, most consumed hard alcoholic drinks like whisky (49%) and arrack (35%). One in ten consumed beer (10%) and about 2% consumed locally brewed illicit alcohol (neera, kalabatti). In rural areas, arrack was the most commonly consumed alcoholic beverage (52%) while whisky was more frequent in urban (63%), town (56%) and slum areas (51%). Those consuming beer was more in the urban areas (23%) compared to other areas. Interestingly, majority of the alcohol users preferred to consume only one type of alcohol (94%) and not change the type of alcohol consumed.

**Table 1:** Patterns of alcohol use

	Rural (*N*=954)		Slum (*N*=1029)		Town (*N*=922)		Urban (*N*=345)		Total	
	No.	%	No.	%	No.	%	No.	%	No.	%
Type of alcohol consumed										
Whisky	316	33.1	524	50.9	524	56.8	218	63.2	1582	48.7
Arrack	505	52.9	300	29.2	316	34.3	19	5.5	1140	35.1
Beer	51	5.4	123	11.9	64	6.9	79	22.9	317	9.8
Rum	48	5.0	61	5.9	27	2.9	30	8.7	166	5.1
Brandy	43	4.5	63	6.1	17	1.8	15	4.4	138	4.2
Note: Those consuming Neera (61), Kallabatti (18) and Mixed (0.3) constituted less than 2% of the study population										
Duration of alcohol consumption									
<1 year	6	0.6	2	0.2	6	0.7	-	-	14	0.4
1–5 years	171	17.9	168	16.33	133	14.4	69	20.0	541	16.7
5–10 years	237	24.8	295	28.7	209	22.7	117	33.9	858	26.4
>10years	619	64.9	607	58.9	616	66.87	172	49.9	2014	61.9
Frequency of alcohol consumption										
Everyday	284	29.7	172	16.7	208	22.5	19	5.5	683	21.0
3-4 /week	139	14.5	205	19.9	114	12.3	32	9.3	490	15.1
1-2/week	227	23.8	402	39.1	303	32.8	137	39.7	1069	32.9
1-3/month	142	14.9	169	16.4	176	19.0	85	24.6	572	17.6
7-11/year	71	7.4	41	3.9	57	6.1	41	11.9	210	6.5
3-6/year	64	6.7	39	3.8	57	6.1	30	8.7	190	5.9
2/year	25	2.6	1	0.1	7	0.7	1	0.3	34	1.1
1/year	2	0.2	0	0.0	0	0.0	0	0.0	2	0.7
Type of drinking										
Infrequent light	220	23.1	174	16.9	193	20.9	92	26.7	679	20.9
Infrequent heavy	20	2.1	37	3.6	47	5.1	35	10.1	139	4.3
Frequent light	615	64.5	611	59.4	618	67.0	164	47.5	2008	61.8
Frequent heavy	99	10.4	207	20.1	64	6.9	54	15.6	424	13.1
Pattern of drinking										
Binge drinking	367	38.5	384	37.3	466	50.5	102	29.6	1319	40.6
Feel the effects of alcohol	376	39.4	634	61.6	595	64.5	173	50.1	1778	54.7
Pathological drinking	273	28.6	296	28.8	190	20.6	66	19.1	825	25.4

Frequency of use [Table 1]: Nearly 85% of users consumed alcohol more than once in a month: one-third consuming everyday or every alternate day and one-third consuming once or twice a week. 45% of the users in the rural areas consumed alcohol daily or every alternate day. One-fourth (23%) of town users consumed alcohol daily, while one-fifth in slum areas (20%) consumed alcohol on alternate days. Greater proportions consumed alcohol on a weekly basis (39% and 33% among slum and town areas respectively).

Duration of drinking [Table 1]: Nearly two-thirds (62%) of the users were long-term users (for more than ten years) and 5% were recent users (within 1 to 2 years). There were more long-term users in the rural (65%) and town (68%) populations.

Pattern of drinking [Table 1]: More than four-fifths (83%) of the users consumed less than five drinks per session and thus could be classified as light drinkers: of these three-fourths (62%) were frequent users. Of the heavy alcohol users (17%), three–fourths (13%) drank frequently. The frequent heavy drinkers were more in the slum (50%) and rural areas (23%), while the infrequent heavy drinkers were more in the urban users (10%). There were more frequent light drinkers in the town population as compared to other areas.

Harmful use of alcohol [Table 1]: Every two out of five persons reported to “binge” alcohol (bouts of heavy drinking over very short periods of time); with nearly a fifth reporting doing so more than once a month. About half (50%) town users reported binge drinking. In one-fourth (26%), during the past year, the use could be classified as pathological and in 6%, it was more than once a month. This pattern of drinking was more evident among the rural and the slum users (29% each). Interestingly, 55% of the population said they continued to consume alcohol till they felt the effects of alcohol (drinking till intoxication) and in 29% this happened more than once a month. 30-40% of them were from the slum and town populations.

Context and reasons for use [Table 2 and 3]: All users remarked that it was extremely common and accepted to consume alcohol on social occasions like festivals, parties or functions. More than 60% of the users reported that they drank alcohol in commercial settings (retail stores, liquor shops, restaurants, bars and pubs). Nearly half of users consumed alcohol at homes and this was common in rural areas (63%). Most often people consumed alcohol when alone (85%). Nearly one-third reported to have consumed alcohol while at work and half (50%) of them were from rural areas. The most common (52%) reason for consuming alcohol was to alleviate pain and to induce sleep which was greater in rural and slum areas (58% each). Habituation and peer pressure (when with friends or in social events/occasions) were said to be the key reasons for alcohol use by 45% and 48% respectively; one-fifth (20%) of the users cited financial and family problems as reasons for alcohol use. Only 10% of the users said that they consumed alcohol as a pleasure seeking activity. A greater proportion of town users stated habituation (57%) and peer pressure (67%) as a key reason for continued alcohol use.

**Table 2:** Circumstances of consuming alcohol and place of residence

	Rural (*n*=954)		Slum (*n*=1029)		Town (*n*=922)		Urban (*n*=345)		Total(*n*=3250)	
	No.	%	No.	%	No.	%	No.	%	No.	%
In own home	601	63.0	487	47.3	389	42.2	102	29.6	1579	48.6
In a bar/pub	263	27.6	438	42.6	359	38.9	193	55.9	1253	38.6
In a retail wine store/liquor shop	614	64.4	582	56.6	625	67.8	139	40.3	1960	60.3
In a restaurant	18	1.9	100	9.7	51	5.5	59	17.1	228	7.0
Others	184	19.3	29	2.8	120	13	4	1.2	337	10.4

**Table 3:** Reasons for consuming alcohol

	Rural (*n*=954)		Slum (*n*=1029)		Town (*n*=922)		Urban (*n*=345)		Total(*n*=3250)	
	No.	%	No.	%	No.	%	No.	%	No.	%
As a habit	394	41.3	427	41.5	526	57.0	104	30.1	1451	44.6
In social company	575	60.3	405	39.4	618	67.0	179	51.8	1553	47.7
Pain /strain/for sleep	553	57.9	594	57.7	388	42.1	155	44.9	1690	52.0
To overcome family and financial problems	197	20.7	204	19.8	231	25.0	28	8.1	660	20.3
Hobby and enjoyment	112	11.7	43	4.2	97	10.5	36	10.4	288	8.8

## Discussion

Alcohol is one of the recognized risk factors for ill-health.(2) The new understanding of the problems related to alcohol use is its greater socio-economic impact than hitherto realized. This is alarming especially with an upward trend in the prevalence of alcohol use over the last two decades.(1) There is growing evidence that apart from the total quantum, the pattern of consumption (frequency of use, drinking to intoxication, binge drinking, chronic use) plays an important role in many of the public health problems (Injuries, violence, etc) consequent to alcohol use.(11) The new paradigms of alcohol use viz., decreasing age at initiation, greater permissibility of social drinking, popularity among women, etc., is increasingly, associated with the processes of globalization, urbanization and migration.(12) A combination of all these proximal and distal factors have made alcohol consumption a common practice, with less understanding on impact of alcohol on health, social and economic areas in the Indian society.

Across the four areas, alcohol use was predominantly a male phenomenon. Hard liquor was the preferred drink with an overwhelming loyalty to a single type of alcohol. Majority of the users were married adults, either illiterate or completed high school, and were unskilled workers. Two-thirds were using alcohol for more than 10 years and one-third drank at least once a week; The typical rural user was a young male, illiterate, doing a job involving hard physical labour with low socio-economic status; preferring to consume heavy alcoholic drinks like arrack on a daily or nearly daily basis and having consumed so for more than ten years, at home or at a retail alcohol outlet. The typical urban user was a young male, literate, doing a skilled job, consuming alcohol at least once a week, preferring beer and consumed it in commercial establishments like restaurants, bars/pubs. Further, it could be posited that pattern of alcohol use among urban users mimic a greater extent the town users while slum users mirror a greater extent the rural users albeit with differences in individual patterns.

Studies on alcohol use in India have varied in their methodology and also used different definitions for alcohol use.(7) Adopting the WHO definition, the crude prevalence of alcohol use in the present study was 13%. An earlier study on health behaviours undertaken in four geographic communities observed the prevalence rate of habitual alcohol use among 15–55 years to be 9%.(13) National Family Health Survey - 2 reported a prevalence of 9.6%, while, NFHS 3 estimated the prevalence to be 13.4% among the 15 to 49 year old indicating an increase of 40% in about 7 to 8 years.(14,15) The National Household Survey reported that 26% of male non-institutionalized people in 12 to 60 years ever used alcohol and an estimated 21.4% were current users.(5) The study from Goa found that while 49% of adults consumed alcohol, the proportion of heavy drinking increased with age and was maximum at 40 years of age.(16) These studies definitely indicate to the alarming growth of alcohol use in the country.

Age and gender difference in alcohol use is well documented. The meta analysis by Reddy and Chandrashekar revealed a 10 fold difference in rates between men and women (men 11.9 /1000 population and women 1.7/1000 population).(17) Almost all studies have reported higher use rates among men varying from 26 % to 72 %.(16,18–26) NFHS 3 has reported that one third of men (32%) consumed alcohol as against 2% among women and marginal difference in prevalence for alcohol use among males in urban and rural areas.(15) Several studies have also reported geographic differences in alcohol consumption with rural and urban rates ranging from 2 to 60% and 4.8 to 250 per 1000 respectively.(18–19,21–23,27–30) The huge variations can be attributed to the different types of instruments used, methodologies adopted and the different definitions of alcohol use and interestingly, very few have been done in different populations using similar methodology. Notwithstanding these limitations, using the metaanalysis approach, Reddy and Chandrashekar observed a greater prevalence in rural areas in comparison to urban areas (7.3 v/s 5.8 /1000 population) against the overall prevalence of alcoholism (6.9/1000) in the country.(17) The NFHS 3(15) and our study observed that information on alcohol use and patterns is scanty from transitional areas especially, slums and towns, even though anecdotal evidence exists regarding the greater use of alcohol than formally documented.(31) Mohan *et al*. reported that 26% of the urban slum dwellers abused substances chiefly alcohol in slums of Delhi, as against the present study estimates of 12% in Bangalore city.(32) Apart from age, sex and place of residence, other factors such as caste, education and standard of living also independently influence alcohol use in India.(33)

Depending on patterns of consumption and impact on health, effects of alcohol are termed as hazardous, harmful or not detrimental. Globally, key patterns of alcohol use referred include chronic use, daily or near daily use, bouts of heavy drinking over very short periods of time (aka binge drinking), solitary drinking, drinking in public places, etc.,(11) A cross-cultural comparisons of alcohol consumption in India, Mexico and Nigeria, showed that no single definition of ‘normal drinking’, ‘problem drinking’ or’ alcohol dependence’ applies across all cultures or countries.(34) Notwithstanding this, in the present study, a major pattern observed across the four areas was chronic use and preference for hard liquor. The slum population had similar rates of pathological drinking as rural populace and the town dwellers had rates similar to urban dwellers. In rural areas, alcohol use was frequent and heavy, while in urban areas it was infrequent but heavy. The public health implication for these two different patterns can be visualized as belonging to two ends of the spectrum for management of alcohol-related problems. Thus, in rural areas there would be more number of alcohol-related chronic health problems, while in urban areas intoxication-related problems are likely to predominate. The transitional areas which recorded more of binge drinking, greater proportions of drinking till intoxication and frequent - light drinking would have as its consequence greater acute health problems.

Further, more hazardous patterns emerge with a combination of the basic patterns of use. The emerging trends include: initiation at an early age, greater consumption among women and increasing preference for drinks with high alcohol content. Despite beer consumption contributing to less than 5% of the total alcohol consumed in the country, nearly two-thirds of the available beer are strong beers with strengths greater than 8% v/v.(35)

Alcohol use is considered as a negative coping strategy on a socio-developmental paradigm. In addition, peer pressure (drinking along with friends) has been a significant influencer in continued use of alcohol. Peer pressure and family acceptance (family members getting together for a drink) in the context of the identified patterns of use pose further hazard acutely and also on long-term basis. This could possibly explain the greater proportions of chronic use or binge drinking among the town population or the frequent heavy drinking in slums.

The common thread running through the above patterns of use, which are detrimental to health, social and psychological well-being and societal well-being, is the unrestricted availability and easy accessibility to alcohol. Global evidence indicates restricting supply with respect to age, number of drinks, context of use contribute to reducing harm from alcohol use; stricter enforcement of legislations pertaining to drinking and driving, interpersonal violence, density of retail vendors reduce the demand and acute consequences of alcohol use.(36) In India, the economic returns from alcohol override public health issues with the situation being one of “gaining less and losing more”: the total excise revenue from alcohol from the country during 2004 was lesser than the out of pocket expense consequent to alcohol use (214 million INR v/s 244 million INR).(3) In addition, the process of globalization has influenced both the supply side and the demand side of alcohol. While GATT (General Agreement on Trade and Tariff) had succeeded in reducing tariff, thereby making alcohol cheaper(37) and more widely available as a commercial product, the affluence consequent to globalization has brought in increased consumption and greater acceptability of alcohol use well with its attendant hazards. Amidst this complex scenario, in India, the absence of a public health driven alcohol control policy and the lack of appropriate services for preventing alcohol use is glaring and evident.

In conclusion, the present study is the first study which has looked at patterns of use in four geographic settings and has highlighted the emerging problems. Many of the patterns identified are detrimental to health and potentially cause harm: both immediately and over a period of time (long-term effects). In the absence of a public health driven alcohol policy, waxing and waning implementation of existing legislation and greater influence of the process of globalization, urbanization and migration, the problems due to alcohol use are bound to increase in the coming decades. The present study indicates the need for differential strategies to prevent and control alcohol use problems particularly in transitional and under-privileged areas.
